# SMART-LAMP: A Smartphone-Operated Handheld Device for Real-Time Colorimetric Point-of-Care Diagnosis of Infectious Diseases via Loop-Mediated Isothermal Amplification

**DOI:** 10.3390/bios12060424

**Published:** 2022-06-16

**Authors:** Juan García-Bernalt Diego, Pedro Fernández-Soto, Sergio Márquez-Sánchez, Daniel Santos Santos, Begoña Febrer-Sendra, Beatriz Crego-Vicente, Juan Luis Muñoz-Bellido, Moncef Belhassen-García, Juan M. Corchado Rodríguez, Antonio Muro

**Affiliations:** 1 Infectious and Tropical Diseases Research Group (e-INTRO), Biomedical Research Institute of Salamanca-Research Centre for Tropical Diseases at the University of Salamanca (IBSAL-CIETUS), Faculty of Pharmacy, University of Salamanca, 37007 Salamanca, Spain; juanbernalt95@usal.es (J.G.-B.D.); begofebrer@usal.es (B.F.-S.); beatrizcregovic@usal.es (B.C.-V.); 2 BISITE Research Group, University of Salamanca, Calle Espejo s/n. Edificio Multiusos I+D+i, 37007 Salamanca, Spain; smarquez@usal.es (S.M.-S.); daniel_santos@usal.es (D.S.S.); jm@corchado.net (J.M.C.R.); 3 Air Institute, IoT Digital Innovation Hub (Spain), 37188 Salamanca, Spain; 4 Microbiology and Parasitology Service, Complejo Asistencial Universitario de Salamanca, University of Salamanca, 37007 Salamanca, Spain; jlmubel@usal.es; 5 Internal Medicine Service, Infectious Diseases Section, Complejo Asistencial Universitario de Salamanca, University of Salamanca, 37007 Salamanca, Spain; belhassen@usal.es

**Keywords:** loop-mediated isothermal amplification, point-of-care diagnostics, infectious diseases SARS-CoV-2, mHealth, Neglected Tropical Diseases

## Abstract

Nucleic acid amplification diagnostics offer outstanding features of sensitivity and specificity. However, they still lack speed and robustness, require extensive infrastructure, and are neither affordable nor user-friendly. Thus, they have not been extensively applied in point-of-care diagnostics, particularly in low-resource settings. In this work, we have combined the loop-mediated isothermal amplification (LAMP) technology with a handheld portable device (SMART-LAMP) developed to perform real-time isothermal nucleic acid amplification reactions, based on simple colorimetric measurements, all of which are Bluetooth-controlled by a dedicated smartphone app. We have validated its diagnostic utility regarding different infectious diseases, including Schistosomiasis, Strongyloidiasis, and COVID-19, and analyzed clinical samples from suspected COVID-19 patients. Finally, we have proved that the combination of long-term stabilized LAMP master mixes, stored and transported at room temperature with our developed SMART-LAMP device, provides an improvement towards true point-of-care diagnosis of infectious diseases in settings with limited infrastructure. Our proposal could be easily adapted to the diagnosis of other infectious diseases.

## 1. Introduction

The lack of affordable and simple molecular diagnostic tools for infectious diseases represents a long standing bottleneck in the health improvement of developing countries [1]. This problem has been tackled through the development of point-of-care tests (POCTs), defined as the rapid detection of analytes near the patient to enable better diagnosis, monitoring, and management of diseases [2]. Nowadays, some forms of POCTs have been reported for many Neglected Tropical Diseases (NTDs), including schistosomiasis (rapid tests for cathodic and anodic circulating antigens), as well as strongyloidiasis and other soil-transmitted helminthiasis (microscopy tool Kankanet) [3]. Furthermore, since the advent of the COVID-19 pandemic, the importance of these POCTs has also been highlighted in developed countries. As of April, 2022, close to five billion molecular tests have been performed for the detection of SARS-CoV-2 [4] and, in an effort to overcome that massive technical challenge, over 400 different rapid tests have already been developed [5].

Ideally, POCT tools should be cheap, portable, simple, fast and capable of quantification [6]. The benchmark to assess those features is the ASSURED criterion (Affordable, Sensitive, Specific, User-friendly, Rapid and Robust, Equipment-free, Deliverable) defined in 2004 by the World Health Organization Special Program for Research and Training in Tropical Disease (WHO/TDR) [7]. This criterion has been recently updated to REASSURED, including Real-time connectivity and Ease of specimen collection [8].

A comparison of the main POCTs for the diagnosis of infectious diseases, namely nucleic acid amplification tests (NAATs), antigen detection and antibody detection, reveals that NAATs present the highest sensitivity and specificity [9]. However, they exhibit poor levels of rapidness and robustness, they usually require equipment, and are not affordable nor user-friendly [8]. Alternatively, several isothermal amplification techniques have been developed. Amongst them, LAMP technology is the most widely used alternative. The LAMP method is a one-step amplification reaction based on auto-cycling strand displacement DNA synthesis performed under isothermal conditions (60–65 °C for 45–60 min) in the presence of a *Bst* polymerase using four primers (two inner and two outer primers). The LAMP method specifically recognizes six distinct sequences in target DNA, thus ensuring high specificity, efficiency and rapidity for amplification [10]. Additionally, reverse-transcription LAMP (RT-LAMP) amplifies DNA from an RNA target in a one-step reaction by directly adding a dedicated reverse transcriptase (RTase) or a DNA polymerase with RTase activity to the reaction mixture sharing all benefits of LAMP technology [11]. LAMP, together with other isothermal amplification techniques, overcomes some of the shortcomings classic NAATs present, particularly speed and price limitations. Nevertheless, other limitations, such as user-friendliness, equipment or deliverability, need supportive technology to be fulfilled [12].

A relatively new approach to tackle those limitations is the combination of POCT with mobile health (mHealth), defined by the WHO Global Observatory as “medical and public health practice supported by mobile devices, such as mobile phones, patient monitoring devices, personal digital assistants (PDAs), and other wireless devices” [13]. Combined with diagnostic devices, mHealth can improve the diagnostic and control strategies of infectious diseases [14]. Additionally, the worldwide increase in smartphone adoption, which had already reached 67% of the global population by 2020 and is expected to reach 70% by 2025 [15], could democratize access to mHealth tools.

Various examples of the combination of mHealth devices and LAMP assays have already been presented. Most of these approaches combine an integrated platform to perform the LAMP reaction, usually in the form of a microfluidic cassette, and a smartphone as a readout tool to evaluate fluorescence generated upon amplification [16,17,18,19,20]. Accurate quantification has been achieved through mathematical processing of the signal detected by smartphone cameras [21]. A few examples make use of microfluidic designs combined with colorimetric detection via the RGB (red, green and blue) color sensors of the smartphone camera. This colorimetric detection of amplification is based on Mg^2+^ dependent indicator hydroxynaphthol blue [22,23], thus considerably simplifying the result analysis and cheapening the overall costs of the test. Accurate quantification of genomic DNA has also been achieved, although no patient samples have been examined [22]. Notwithstanding this, other approaches, such as redox indicators [24], lateral-flow assays [25] or centrifugal displays [26], have also been combined with LAMP. Microfluidic designs present the main advantage of the integration of the various steps of a multistep diagnostic assay, including sample handling, processing as well as signal amplification and detection [27]. Although they show a promising future, they also pose numerous questions regarding the following: automation (most assays require extensive user intervention for sample preparation prior to amplification, as well as for reagent addition to the diagnostic platform); the need to improve sensitivity, selectivity and stability of sensing moduli; scalability and clinical validation [28].

Attending to some of those limitations, in this work we present the SMART-LAMP, a handheld portable device to perform real-time isothermal amplification reactions, based on simple colorimetric measurements in standard 0.2 mL Eppendorf tubes, controlled via Bluetooth by a dedicated SMART-LAMP app on a smartphone. We show its applicability as a POCT tool using stabilized reaction mixes that can be stored and transported at room temperature (RT) for extended periods of time. We tested the applicability of SMART-LAMP for the detection of different infectious agents: *Schistosoma mansoni, S. haematobium, Strongyloides* spp. and SARS-CoV-2. Additionally, we tested the diagnostic utility of SMART-LAMP on patients suspected of having COVID-19.

## 2. Materials and Methods

### 2.1. Human Samples

The study protocol was approved by the Clinical Research Ethics Committee of Investigation with Drugs of the University Hospital of Salamanca, Spain (CEIMC 2020.06.530) on 19 October 2020. The procedures described here were carried out in accordance with the ethical standards described in the Revised Declaration of Helsinki in 2013. All patient data were anonymized and unidentified.

### 2.2. DNA and RNA Purification

For amplification trials, genomic DNA (gDNA) from *Schistosoma mansoni, S. haematobium* and *Strongyloides venezuelensis,* as well as SARS-CoV-2 RNA samples from positive COVID-19 patients, were used. DNA from *S. mansoni* and *S. haematobium* was obtained from freeze-stored adult worms. DNA from *S. venezuelensis* was purified from freeze-stored infective third-stage larvae (iL3). DNA was extracted using the NucleoSpin Tissue Kit (Macherey-Nagel, GmH & Co., Düren, Germany) following the manufacturers’ instructions. Purified DNA was measured in triplicate using a Nanodrop ND-1000 spectrophotometer (Nanodrop Technologies, Wilmington, DE, USA), and then diluted in ultrapure water to a final concentration of 5 ng/μL. 

SARS-CoV-2 RNA samples were obtained from nasopharyngeal swabs from suspected COVID-19 cases collected and stored in Sample Preservation Solution (MOLE BIOSCIENCE, SUNGO Europe B.V., Amsterdam, The Netherlands) as part of the routine testing of patients for COVID-19 at the University Hospital of Salamanca (Salamanca, Spain). Swabs were transported to the e-INTRO group laboratory and processed in a biosafety level 2 cabin until inactivation by mixing with the lysis buffer. Afterwards, RNA was isolated using NZY Viral RNA Isolation Kit (NZYTECH, Lisbon, Portugal), following manufacturers’ instructions.

### 2.3. Fresh-LAMP and Fresh-RT-LAMP Assays

For LAMP assays, we used the set of primers and reaction conditions previously described by our group for DNA detection of *S. mansoni* [29], *S. haematobium* [30], *Strongyloides* spp. [31] and SARS-CoV-2 RNA detection [32]. In brief, fresh-LAMP reaction mixtures (15 μL) contained 1.6 M FIP/BIP primers, 0.2 μM F3/B3 primers, 0.4 μM LF/LB primers (if applicable), 1.4 mM of each dNTP, (BIORON GmBH, Römerberg, Germany), 6 mM MgSO_4_, and 1× Isothermal Buffer (20 mM Tris-HCl (pH 8.8), 50 mM KCl, 10 mM (NH_4_)_2_SO_4_, 2 mM MgSO_4_, 0.1% Tween20) for Bst 2.0 Warm Start (WS) (0.32 U/μL) (NEW ENGLAND BIOLABS Ltd., Ipswich, MA, USA). Reactions were performed at 65 °C for up to 1 h. Fresh-RT-LAMP reactions for SARS-CoV-2 contained the same reagents and concentrations as fresh-LAMP, with an additional WS RTx Reverse Transcriptase enzyme (0.3 μL) (NEW ENGLAND BIOLABS Ltd., Ipswich, MA, USA). Reactions were performed at 63 °C for up to 40 min.

Amplification detection was performed via real-time fluorescence monitoring or real-time RGB color monitoring. Real-time fluorescence-based reactions were performed either in the portable Genie III instrument (OPTIGENE, Horsham, UK) or the PCRmax ECO 48 real time PCR system (PCRmax, Stone, UK) using 0.24 μL of EvaGreen (EG) 20× dye (BIOTIUM, Inc, Fremont, CA, USA). For real-time colorimetric-based reactions, performed in the SMART-LAMP prototypes, 0.008% *w*/*v* of malachite green (MG) (Sigma-Aldrich, San Luis, CA, USA) was included in the master mixes. In all tests, 2 μL of nucleic acid template (DNA or RNA) for the positive control (PTC), or ultrapure water for the negative control (NTC), were added to the reaction mixture.

### 2.4. Dry-LAMP: Stabilization of LAMP Reaction Components for Ready-to-Use Tests

Dry-LAMP (for DNA detection) and dry-RT-LAMP (for RNA detection) reaction mixes were obtained by desiccation of fresh-LAMP mixes in the presence of 2M trehalose (Sigma-Aldrich, San Luis, CA, USA) using a protocol previously described by our group [33] and later improved [32]. Briefly, fresh master mixes were dried separately in two partial mixes. One was placed in the bottom of the tubes containing the primer mix, dNTPs, *Bst* 2.0 WS and *RTx* WS (if applicable) with 1.8 μL of 2M trehalose. The other was pipetted in the tube caps, containing isothermal buffer, additional MgSO_4_, and the dye (MG or EG) in the presence of 2.25 μL of 2M trehalose. Then, the stabilization consisted of a 30 min single-step desiccation protocol in a Concentrator Plus (Eppendorf, Hamburg, Germany) at RT. This procedure did not require lyophilization, greatly reducing the technical requirements.

Subsequently, desiccated reaction mixes were stored protected from the light at RT until use. Upon rehydration, tubes were placed upside down for 2 min to accomplish full reconstitution of the master mix before the reaction started.

Reactions were performed under the same conditions as the fresh-LAMP reaction. Amplification was also detected by the same strategies. To carry out the dry-LAMP assays, reaction times for *S. mansoni* and *Strongyloides* spp. were increased to 90 min, while reaction times for dry-RT-LAMP SARS-CoV-2 were increased to 50 min. For *S. haematobium* amplification, the reaction time remained 60 min long in both fresh- and dry-LAMP formats.

### 2.5. SMART-LAMP: Principal Modules and Characteristics

The SMART-LAMP device comprises a 3D-printed handheld platform manufactured with Fused Deposition Modelling (FDM) technology in polylactide (PLA) material for the simultaneous incubation and real-time data acquisition of eight samples. It has a modular design which comprises: (i) a sample incubation module, (ii) a sample reading module and, (iii) a transmission module.

The *sample incubation module* maintains the sample at 60–70 °C for a period of up to 120 min. Intelligent algorithms were designed to allow this module to modify both the temperature (ranging from 40 °C to 80 °C) and the processing time (ranging from 5 to 120 min). It also includes LED and acoustic indicators that provide information about the device status (such as “not ready”, “ready to use”, “connected” or “in-use”), temperature and the elapsed and remaining reaction times. To stabilize the temperature a Proportional Integral and Derivative temperature control algorithm (PID) was applied [34] maintaining the temperature with a ±0.9 °C accuracy. In brief, the PID was implemented through the PID_v1 library of Brett Beauregard [35] with values of Kp = 4, Ki = 1 and Kd = 1, adjusted via trial and error. Due to the thermal damping caused by the thermo-block and its isolation, the PID was deactivated when the temperature reached the target. Temperature maintenance was achieved manually to relieve the microcontroller unit (MCU) from computational load, as results were the same as the ones obtained via PID control. Temperature is calculated with the Steinhart-Hart equation. Three measurements were realized to obtain A, B and C coefficients of the equation. The system continuously monitors the temperature to ensure safety and prevent fires, whereby audible alarms are triggered via software in the event of excessive or uncontrolled temperature rises.

The *sample reading module* consists of photoresistors and fiber optics elements. A TCS3472 color sensor was used as detector, which provides a digital return of red, green, blue (RGB), and clear light sensing values. The high sensitivity, wide dynamic range, and infrared filter (IR Block filter) make the TCS3472 an ideal color sensor solution for use under varying lighting conditions and through attenuating materials. Additionally, overhead white LED lights were located over the samples providing the right light for effective reading.

The *transmission module* includes four systems necessary for the reception and protection of the sample results and other relevant information: data reception, data protection, transmission and storage. Regarding data reception, along with the results of the sample, the system incorporates other complementary information related to the context in which the sample is taken, such as GPS position, information about the patient, healthcare professional responsible for taking sample, measurement equipment information, incidents, etc. The information is stored in compliance with the HL7 specifications [36] Data protection includes all the elements needed to encrypt the data, safeguard the identity of the users, and certify both the origin and the correction thereof, so that all international data protection laws are complied with. In terms of transmission, the virtual organization facilitates communication through Bluetooth Low Energy (BLE) to send data to a connected device, following the standards of the Edge Computing architecture. Where storage is concerned, the module stores the data collected by means of an internal Process Context Block (PCB) memory to facilitate delayed retransmission, in case there is no signal coverage [37]. 

In addition to the three fundamental modules, SMART-LAMP is designed to be used in areas with and without an internet or electrical connection through two main features: (i) the generation and recharge of the battery. The device uses MC73831T and three Li-ion batteries (NCR186508), chosen to power the entire system. They render well in terms of discharge capacity and in the number of possible recharges [38] and (ii) SMART-LAMP Interaction Interface, which allows the device to visualize and validate the results, send information, backups, etc. The interface is designed both at the level of the data measurement system, and at the level of the analysis and information storage system. The generated information can be visualized on personal computers as well as on tablets or smartphones, and is adaptable to different screen formats. The visualization is also displayed on the SMART-LAMP device on an alphanumeric LCD screen with 16 characters and 2 lines. The interface may analyze the information considering geographical, local and/or personal parameters. In addition, a mobile application has been created to display the results in real time through a friendly interface on the iOS (Apple Inc., Los Altos, CA, USA) operating system, where measurement data can be accessed. The application also allows interaction with the SMART-LAMP device, starting or forcing the stop of a measurement, as well as registering a user, linking to a nearby device and monitoring the battery level of the device. Although the smartphone app is not yet publicly available, it will be ready for download in the near future via the website https://smartlamp.es/en (accessed on 7 June 2022).

Single-line circuit sketches of SMART-LAMP components are shown in Figure S1 and printed circuit boards in Figure S2.

### 2.6. SMART-Lamp Assessment

#### 2.6.1. Color Readout and Temperature Profile

For color readout assessment, 2-fold serial dilutions of MG dye in water were measured with the three SMART-LAMP prototypes, ranging from 0.1% *w*/*v* to 7.81 × 10^−4^% *w*/*v*. Each dilution was measured in triplicate in each well of each prototype.

The temperature profile was assessed by measuring the ramping times of the device between RT and reaction temperature (25 °C to 65 °C), between reaction temperature and inhibition temperature (65 °C to 80 °C), and back to reaction temperature (80 °C to 65 °C). To evaluate the influence of the batteries in the ramping times, measurements with 100%, 75%, 50%, 25%, and under 25% of battery charge were taken. Temperature stability was assessed for 1 h at 65 °C, with measurements taken every 5 min.

#### 2.6.2. Positive and Negative Predictive Values

Four positive controls (PTCs) and four negative controls (NTCs) from each fresh-LAMP and dry-LAMP assay were used to establish the best measurement to discriminate positive and negative results. RGB values were monitored at real-time with the SMART-LAMP. Absolute values of each component at the end of the reaction (t_final_), as well as relative differences between color components at t_final_ and reaction times 0 min (t_0_), 5 min (t_5_), 10 min (t_10_), and 15 min (t_15_) were considered.

Once the most appropriate measurement was established, 40 controls (20 PTCs and 20 NTCs) were tested for each dry-LAMP to establish the positivity threshold value and to calculate positive predictive value (PPV) and negative predictive value (NPV) for the SMART-LAMP. To account for any possible effect of long-term room-temperature storage of dried mixes, 20 controls (10 PTCs and 10 NTCs) were tested after >30-days of room-temperature storage.

#### 2.6.3. Analytical Sensitivity

To assess the sensitivity of dry-LAMP mixes, 10-fold serial dilutions, starting from 5 ng/μL of each template, were prepared and tested, both with EG in the Genie III device and with MG as dyes in the SMART-LAMP prototypes. For each LAMP assay, dilutions were prepared down to the limit of detection described in their original descriptions: 10 fg in the case of *S. mansoni* and *S. haematobium* [29,30], and 10 pg for *Strongyloides* spp. [31]. For LAMP amplification of SARS-CoV-2, RNA from a patient sample with a RT-qPCR Ct value of 25, for the ORF1ab region, was used as a PTC; similarly, 10-fold serial dilutions, down to the 1:100 limit of detection originally described [30], were prepared to assess sensitivity.

### 2.7. Proof of Concept: COVID-19 Patients Sample Analysis

Eighty RNA samples from patients with symptoms compatible with COVID-19 disease (20 negative and 60 positive results via RT-qPCR) were used as a proof of concept of the applicability of the SMART-LAMP for diagnostic purposes. For comparison, the 80 samples were first analyzed by RT-qPCR in a PCRmax ECO 48 real time PCR system using the SARS-CoV-2 One-Step RT-PCR Kit–IVD (Nzytech, Lisbon, Portugal), following manufacturers’ instructions. The 80 RNA samples were also tested by fresh-RT-qLAMP and dry-RT-qLAMP in the Genie III instrument and dry-RT-qLAMP in the SMART-LAMP prototypes. Correlation of positive results between the RT-qPCR kit and the three different LAMP protocols were measured and evaluated via Pearson’s correlation.

### 2.8. Statistical Analysis

Statistical analysis was performed in R (version 3.6.3). Packages ggplot2, ggbeeswarm, ggpubr, grid, EnvStats and dplyr were used to analyze and visualize the data. Comparisons among the three SMART-LAMP prototypes were performed via one-way analysis of variance (ANOVA). Comparisons between positive and negative values were evaluated via the Mann-Whitney U test. Correlations assays were performed via Pearson’s correlation. 

## 3. Results and Discussion

### 3.1. Device Design and Construction

**Figure 1:**
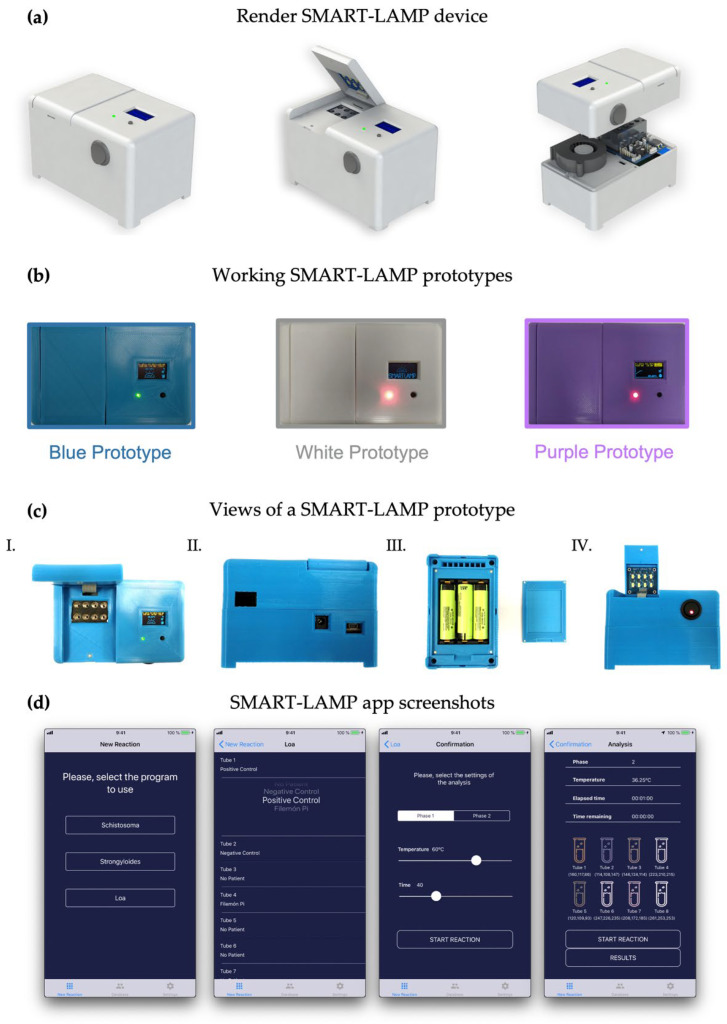
Design and principal features of the SMART-LAMP and SMART-LAMP app: (**a**) Render of the SMART-LAMP prototype with SolidWorks. From left to right: closed SMART-LAMP; SMART-LAMP with the lid of the incubation module opened; and internal structure of the SMART-LAMP prototype; (**b**) Top view of the three working SMART-LAMP prototypes used; (**c**) Different views of the one SMART-LAMP device: I. Top view of the SMART-LAMP device with the incubation module lid opened; II. Posterior view with all ports and device connections exposed, from left to right: fan opening, power supply port and USB port for computer connection; III. Inferior view with the exposed MC73831T and three Li-ion batteries that power the SMART-LAMP device. To the right, the lid used to cover the batteries which is magnetically attached to the device for easy removal and reinstalment; IV. Frontal view of the SMART-LAMP with the lid opened, exposing the overhead with LED used to illuminate samples while RGB measures are taken; (**d**) SMART-LAMP app screenshots. From left to right: reaction program selection, sample identification, duration and temperature setup through sliding buttons, interface of the SMART-LAMP app while a reaction is being carried out, showing the temperature of the device, the time elapsed and the RGB measurements of each well.

The SMART-LAMP was digitally designed with SolidWorks, which yielded a render of the prototype that can be observed in Figure 1a. The prototype cases were 3D-printed and manually assembled with all the necessary electronic components. The SMART-LAMP prototypes presented dimensions of 12.5 cm × 7.8 cm × 8.8 cm and 850 g of weight (including three NCR186508 power batteries).

This design proved to be robust enough to withstand temperatures above 80 °C without any sign of deterioration in the three different constructed prototypes. Battery life was found to be 8–9 h at reaction temperature (65 °C) which, for a typical 1 h reaction, would allow for 7 to 8 reactions before battery replenishing. The sample incubation module, encompassing a heating block, was designed to simultaneously fit eight universal 0.2 mL Eppendorf tubes (see Figure 1b,c). The heating of the sample block was achieved by two polyamide heaters of 4.8 W at 12 V. The block was thermically isolated with a rock wool covering. In addition, to protect the device case, batteries and all the SMART-LAMP components, a fan was activated to extract hot air from the interior of the devices.

The production cost of the device has been estimated to be around €300. A list of all necessary materials for building the device can be found in Table S1.

All three prototypes efficiently connected via Bluetooth to both an iPad Air and an iPhone 11. The SMART-LAMP iOS app allowed for an easy selection of reaction conditions and a continuous monitoring of RGB values of each well. At the end of the reaction, the researcher could export all data in csv format, as well as revise the color profile of each tube within the app (Figure 1d and Figure S3). The time of reaction and GPS location could also be extracted. A patient database could be created within the app to store clinically relevant data from the patients.

Other demonstrations of devices performing isothermal amplification can be found in the literature. However, most of them are based on intricate chip or microfluidic designs and use fluorescence as a measurement of amplification [16,17,18,19,20].

Despite lacking a microfluidic design, the SMART-LAMP is still compact and even smaller than other devices already described for LAMP [39]. The simple structure of SMART-LAMP, operated like a regular thermo-block, avoids complex schemes that often hamper the deployment of microfluidic diagnostic tests [40]. 

### 3.2. Color Readout and Temperature Profile Assessment of the SMART-LAMP

**Figure 2:**
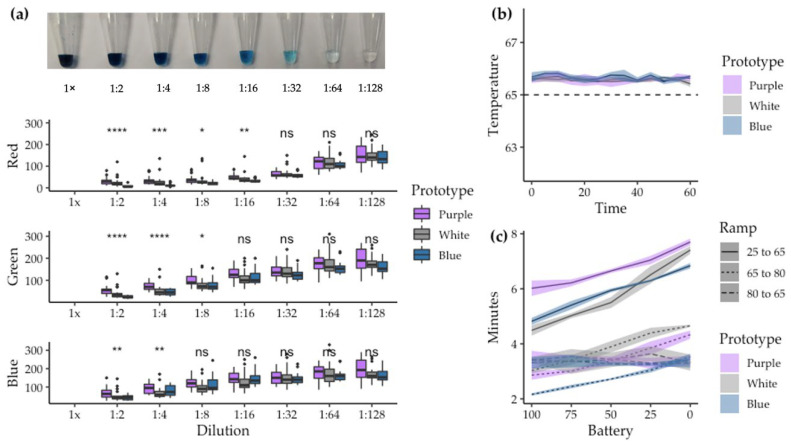
Basic measurements of color readout and temperature profile of the SMART-LAMP devices: (**a**) Color readout of the SMART-LAMP with MG dilutions in water. In the top picture, 15 μL of MG dilutions in water in 0.2 mL Eppendorf tubes are presented, ranging from a 0.1% *w*/*v* dilution (1×) down to 7.81 × 10^−4^% *w*/*v* (1:128), in a 2-fold serial dilution fashion. Boxplots representing RGB values for all eight dilutions are presented (from top to bottom: Red component, Green component and Blue component), measured in triplets in each well of each prototype. ANOVA-test results comparing the three prototypes are presented. (n/s: non-significant, *: *p*-value ≤ 0.05, **: *p*-value ≤ 0.01, ***: *p*-value ≤ 0.001, ****: *p*-value ≤ 0.0001). (**b**) Temperature profile for one hour reaction at 65 °C in each prototype. Measured in triplets. (**c**) Ramping times to achieve reaction temperature (65 °C) from room temperature (25 °C), from reaction temperature to inhibition temperature (80 °C) and from inhibition temperature back to reaction temperature. Measured for different battery percentages. Each measurement was taken in triplicates.

Two-fold serial dilutions of malachite green (MG) dye in water were measured with the three SMART-LAMP devices, ranging from 0.1% *w*/*v* to 7.81 × 10^−4^% *w*/*v*. As expected, higher concentrations of the dye resulted in lower RGB values and lower concentrations in higher RGB values. Variability among measurements in each device increased as the dye concentration decreased. However, differences among devices were significant at high MG concentrations, while they were not at low concentrations (Figure 2a). The low concentrations resulted in colors that were the most similar to the ones obtained in real LAMP reactions. No statistically significant differences were found among the devices for those dilutions; thus, the following experimentation used the three devices indistinctively. Moreover, absolute RGB values were not considered a good indicator of DNA amplification, due to the high variability detected in color readout.

Focusing on the temperature profile, SMART-LAMP was able to rise from RT (25 °C) to reaction temperature (65 °C) in 4–8 min depending on the battery percentage of the working prototype. To inhibit the enzymatic activity at the end of the LAMP reaction, the SMART-LAMP was able to rise from reaction temperature (65 °C) to inhibition temperature (80 °C) in 2–5 min depending on the battery percentage. Although the inhibition temperature was not necessary in real-time amplification reactions, it may nevertheless be of interest when subsequent down-stream analysis is required with the amplified products (e.g., electrophoresis, band purification), or for end-point colorimetric visualization of the results. The cooldown of the device from inhibition temperature to reaction temperature was maintained around 3 min, regardless of the battery percentage (Figure 2c). Once the reaction temperature was achieved, it was maintained constant throughout the reaction. In all three prototypes, the temperature never cooled under the predetermined value, and never over-heated by more than 0.9 °C (Figure 2b).

### 3.3. Positive and Negative Predictive Values of SMART-LAMP

To assess the predictive positive capacity of the SMART-LAMP devices, the difference between the final value (t_final_) of RGB components and the values of RGB components at 0 (t_0_), 5 min, 10 min (t_10_) and 15 min (t_15_) after the reaction started were evaluated for both fresh-LAMP and dry-LAMP reactions. For dry-LAMP reactions, stable desiccation of all reaction mixtures was achieved and well attached pellets were formed, both in the cap and at the bottom of the tubes (Figure S4). The pellets remained well-preserved for more than 30 days at RT. 

**Figure 3:**
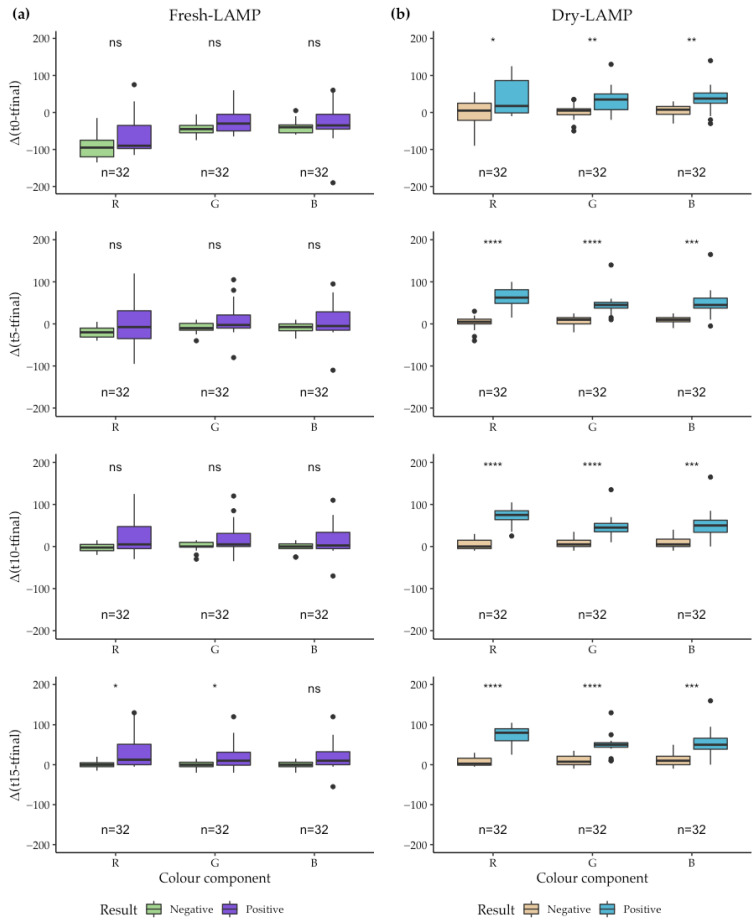
Differences between the final RGB values and the RGB values at 0, 5, 10 and 15 min for fresh-LAMP and dry-LAMP reactions measured by SMART-LAMP devices: (**a**) For fresh-LAMP and (**b**) dry-LAMP a total of 32 LAMP reactions (*n* = 32) were performed: 8 LAMP reactions (including 4 PTC and $ NTC) for *Schistosoma mansoni*, *S. haematobium*, *Strongyloides* spp. and SARS-CoV-2. Mann-Whitney U test: n/s: non-significant, *: *p*-value ≤ 0.05, **: *p*-value ≤ 0.01, ***: *p*-value ≤ 0.001, ****: *p*-value ≤ 0.0001.

Four positive controls (PTC) and four negative controls (NTC) of each LAMP assay were used (*Schistosoma mansoni*, *S. haematobium*, *Strongyloides* spp. and SARS-CoV-2). For each time-point, differences between PTC and NTC were more significant for dry-LAMP than fresh-LAMP assays. For fresh-LAMP assays, the only measurement that resulted in significant differences between positives and negatives (evaluated by the Mann-Whitney U test) was obtained analyzing the red and green components. Specifically, the reduction in the red component between t_15_ and t_final_ presented the most significant difference between positive and negative. For dry-LAMP reactions, the best predictor of amplification was also the reduction in the red component of RGB between times t_10_ or t_15_ and t_final_ (Figure 3). Greater significance was found in dry-LAMP reactions than in fresh-LAMP reactions which correlated with the increased colorimetric variation that could be observed by the naked eye (Figure S4). Given that the fastest amplifications we have detected with dried mixes were close to 15 min, the reduction of the red component of RGB between t_10_ and t_final_ was selected as the predictor of positivity.

**Figure 4:**
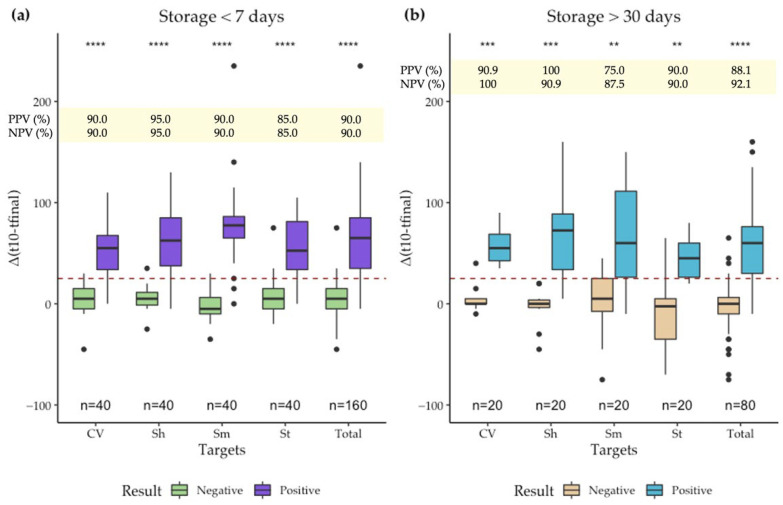
PTC and NTC analyzed with the SMART-LAMP for different dry-LAMP assays and storage times: (**a**) Differences between values of the R component of RGB 10 min after the start of the reaction and final value at reaction-end for different LAMP assays stored less than 7 days at RT after desiccation; (**b**) Differences between values of the R component of RGB 10 min after the start of the reaction and final value at reaction-end for different LAMP assays stored over 30 days at RT after desiccation. In yellow boxes, positive predictive values (PPV) and negative predictive values (NPV) of the SMART-LAMP are indicated. Targets: SARS: SARS-CoV-2; Sh: *S. haematobium*; Sm: *S. mansoni*; St: *Strongyloides* spp. Mann-Whitney U test: n/s: non-significant, **: *p*-value ≤ 0.01, ***: *p*-value ≤ 0.001, ****: *p*-value ≤ 0.0001.

Next, 20 PTCs and 20 NTCs from each dry-LAMP assay were used to establish the positivity threshold value. To do so, 40 dry-LAMP mixes of each LAMP assay were stored at RT and rehydrated within a 7-day period. In all dry-LAMP assays a decrease in the red component of RGB of 25 units between the value at t_10_ (10 min after the start of the reaction) and t_final_ (at the end of the reaction) maximized the positive and negative predictive capacity of the SMART-LAMP (Figure 4). Thus, any difference equal or superior to 25 was considered positive, any difference under 25 was considered negative. Then, to evaluate the effect of long-term storage in color measurements, 20 dry-LAMP mixes were stored for over 30 days at RT, followed by the analysis of 10 PTCs and 10 NTCs in the SMART-LAMP. The results from dry-LAMP mixes stored at RT for less than 7 days were compared to mixes stored for over 30 days, and no significant differences were found between long-term and short-term stored dry-LAMP mixes (Figure 4). Overall, a 90% (CI95: 83.4–96.6) positive predictive value (PPV) and a 90% (CI95: 83.4–96.6) negative predictive value (NPV) were obtained for dry-LAMP mixes stored under 7 days at RT. Storage over longer periods of time did not significantly affect predictive values: 88.1% (CI95: 78.3–97.9) PPV and 92.1 NPV (CI95: 83.5–100). Subtle differences between LAMP assays were detected, with the SMART-LAMP showing slightly better predictive values for the SARS-CoV-2 and *S. haematobium* assays than the *S. mansoni* and *Strongyloides* spp. Thus, the reactions with faster kinetics (SARS-CoV-2 and *S. haematobium*) showed slightly improved PPV and NPV, regardless of the time of storage. The device, as discussed below, has shown good diagnostic capabilities. Nevertheless, predictive values obtained could probably be improved. The primary improvement would come from a reduction in variability of color measurements. This could be achieved via a standardized montage of SMART-LAMP devices. Additionally, although providing a single positivity threshold allows for a straightforward use of the device and stabilized mixes for the diagnosis of new pathogens, it can also limit the predictive values, when compared to individually optimized positivity thresholds.

Conclusively, it was demonstrated that SMART-LAMP enabled the detection of color changes associated with DNA amplification. The device proved to be useful for the detection of different infectious agents: *S. mansoni*, *S. haematobium*, *Strongyloides* spp., and SARS-CoV-2. Moreover, the results evidenced that a single positivity threshold could be used, regardless of the LAMP assay performed. Thus, the adaptation of this device for the diagnosis of other diseases should be foreseeably straightforward. Examples of color-based detection with a smartphone are available in the literature, using hydroxynaphthol blue. Although they are able to perform real-time detection of amplification in a similar timeframe to our SMART-LAMP (60 min) [22,23], they still rely on a microfluidic chip. It is clear that microfluidics offers some advantages over a design such as the SMART-LAMP, particularly in terms of reduced reaction volumes and integration, achieving controlled transport, mixing and reaction in specific microchambers [41]. In addition, some of their challenges, such us clinical validation [28], are shared between both approaches. However, the small scale and sometimes close to single-molecule approaches of microfluidics magnify subtle interactions that can result in inaccurate results. The interactions between plastics, adhesives and other materials can profoundly affect diagnostic performance [42]. This can mean that microfluidic designs are not cost-effective on a large scale, whereas the common laboratory materials used in the SMART-LAMP are more easily acquired, and repaired if, or when, necessary.

### 3.4. Analytical Sensitivity

**Figure 5:**
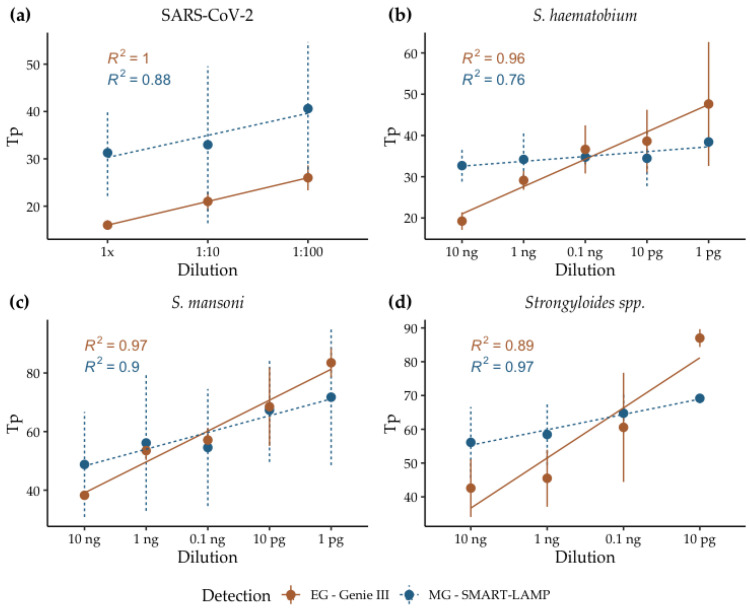
Sensitivity of real-time dry-LAMP assays performed in the Genie III (EG) and the SMART-LAMP (MG) devices: (**a**) SARS-CoV-2; (**b**) *S. haematobium*; (**c**) *S. mansoni*; (**d**) *Strongyloides* spp.

Ten-fold serial dilutions of PTC were analyzed in triplicates for each of the dry-LAMP assays, both measuring fluorescence signal with Eva Green (EG) dye in the commercial Genie III device and color turn of MG via RGB in the SMART-LAMP device (Figure 5). Limit of detection (LoD) was proven to be equal for fluorescence-based detection and RGB-based detection. Sensitivity was not reduced by the stabilization process in the case of dry-LAMP reactions for SARS-CoV-2 and *Strongyloides* spp. amplification [31,32]. However, in the case of dry-LAMP for the amplification of *S. haematobium* and *S. mansoni*, the sensitivity was slightly lower than that obtained in fresh-LAMP reactions: 0.1 pg vs. 10 fg for *S. haematobium* [30] and 1 pg vs. 10 fg for *S. mansoni* [29]. According to previous results obtained by our group [33] and others [43], sensitivity tends to decrease after stabilization, especially after extended periods of storage at room temperature. Those effects have been observed not only in the LAMP reactions, but also in other isothermal amplifications, such as rolling circle amplification (RCA) [44]. In some cases, a reduction in signal intensity may occur, which has been observed after stabilization of the reagents via lyophilization in RT-qPCR [45].

The kinetics of all the reactions correlated well with the analyzed concentrations. The time to positivity (Tp) of the dilutions analyzed by real-time dry-LAMP on the Genie III device fitted linear regression models correctly, with R^2^ coefficients ranging from 1 for SARS-CoV-2 to 0.89 for *Strongyloides* spp. On the other hand, for SMART-LAMP results, linear regression ranged from R^2^ = 0.97 for *Strongyloides* spp. to 0.78 for *S. haematobium.* Of note, for high concentrations of the target, flourescence-based amplification detection was more efficient in all cases. However, at low concentrations, SMART-LAMP could detect amplification earlier than the Genie III device. The reaction time slightly increased when utilizing dried mixes compared to fresh ones. The reasons behind this phenomenon are not clear, but a possible explanation could be the action of the trehalose matrix in retarding conformational dynamics in dehydrated protein systems [46]. In a trehalose system, guest molecules (*Bst* polymerase in this case) are homogeneously integrated into a hydrogen-bond network of water and trehalose, strongly limiting their mobility [47].

Evidently, when comparing EG and MG, an important caveat must be considered, as the amplification monitoring is performed via different mechanisms. While EG directly binds to the DNA [48], MG monitors amplification via the concentration of free Mg^2+^, which is reduced as amplification takes places and it binds to the pyrophosphate liberated [49]. Notwithstanding this consideration, both are valid approaches for amplification monitoring widely used in LAMP. Interestingly, EG has been shown to have an inhibitory effect, that can reduce both reaction rates, as well as final amplification levels [50]. This effect could partly explain the differences observed at low concentrations of template between EG and MG.

### 3.5. Proof of Concept: COVID-19 Patients Sample Analysis

**Figure 6:**
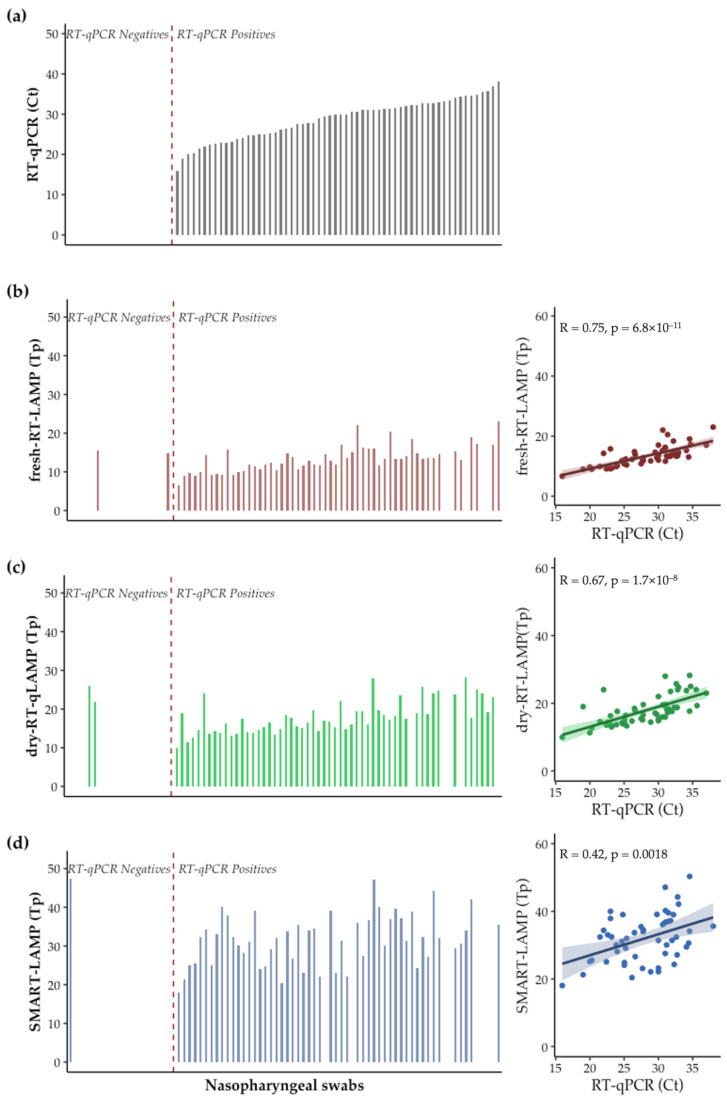
Analysis of RNA samples from patients suspected of having COVID-19. On the left panels, results from the analysis of the 80 samples are shown ordered from low to high RT-qPCR Ct values obtained. On the right panels, correlation plots between Ct and Tp values in RT-qPCR and LAMP assays, respectively, are represented. R and *p* values obtained by Pearson’s correlation are shown in each graph: (**a**) RT-qPCR Ct values of the 80 samples analyzed; (**b**) Fresh-RT-qLAMP Tp values of the 80 analyzed samples and their correlation with RT-qPCR results; (**c**) Dry-RT-qLAMP Tp values of the 80 analyzed samples and their correlation with RT-qPCR results; (**d**) SMART-LAMP Tp values of the 80 samples analyzed and their correlation with RT-qPCR results.

RNA from 80 patients with suspected COVID-19 (20 negative and 60 positive) were analyzed by RT-qPCR, fresh-RT-LAMP, and dry-RT-LAMP (in the commercial device Genie III) as well as in the SMART-LAMP to evaluate the applicability of the device in “real-world” conditions. Taking RT-qPCR results as the diagnostic standard [51], fresh-RT-LAMP showed a sensitivity of 93.3% (CI95: 87.0–99.6) and a specificity of 90.0% (CI95: 76.9–100). No deleterious effect was observed due to the desiccation of the RT-LAMP components, as dry-RT-LAMP showed a sensitivity of 95.0% (CI95%: 89.5–100) and a specificity of 90.0% (76.9–100). A slight decrease in sensitivity was observed when performing the analysis in the SMART-LAMP, resulting in 88.3% (80.2–96.5). Nevertheless, specificity was improved slightly to 95.0% (85.4–100). If only RNA samples with a Ct < 33 value in RT-qPCR, (samples with an estimated viral load over 500 copies [52]) were considered, then sensitivity of fresh-RT-LAMP increased to 100%, that of dry-RT-LAMP to 97.9% and that of SMART-LAMP to 95.9% (Figure 6).

Regarding reaction kinetics, a clear decrease in correlation between RT-qPCR Ct values and LAMP Tp values was observed when using the SMART-LAMP (Pearson’s correlation coefficient; R = 0.42). The decrease in correlation was not a consequence of the desiccation process as fresh-RT-qLAMP and dry-RT-qLAMP assays showed highly similar Pearson’s coefficients (0.75 and 0.67, respectively), and when comparing SMART-LAMP results to either fresh-RT-qLAMP or dry-RT-qLAMP, the Pearson’s coefficient in both cases was 0.45. Still, the SMART-LAMP resulted in a moderate correlation, with a single analysis of the samples (Figure 6b–d).

The sensitivity and specificity obtained in our study were comparable to those obtained with other POCT approaches for SARS-CoV-2 but performed in a much simpler manner. Soares et al. [18] tested 162 nasopharyngeal swabs, collected from patients with COVID-19 symptoms, on a centrifugal microfluidic platform. They detected amplification with a bead-based approach, obtaining a sensitivity of 96.6% for samples with a low Ct (Ct < 26) and a specificity of 100%. In the work of Rodríguez-Manzano et al. [17], a lab-on-chip RT-LAMP assay was developed on the basis of semiconductor technology paired with a smartphone for result visualization. As a benchtop technique, when analyzing 183 clinical samples (including 127 positives) the assay showed a LoD of 10 RNA copies per reaction with 91% sensitivity and 100% specificity. The average Tp was 15.45 ± 4.43 min. By testing a sample subset on the POCT platform developed, the authors showed comparable results with the benchtop instrument, with an average Tp of 12.68 ± 2.56 min for positive samples (*n* = 34). Other approaches have combined a magnetic-based purification of RNA samples with a smart-phone based detection approach, obtaining reduced false positive rates and being able to detect positive samples within large pools of patients bronchoalveolar lavages [53]. Chen et al. [54] developed a 3D-printed portable station to detect amplification at the end of the reaction. They tested 7 positive and 3 negative respiratory swab samples of SARS-CoV-2, obtaining 100% sensitivity and 100% specificity.

In all, we present a novel handheld device for the diagnosis of infectious diseases via colorimetric real-time LAMP assay. Only a few examples of similar platforms have been described, which rely either on pH indicators [55,56] or Hydroxynaphtol blue dye [56], but, to the best of our knowledge, none of them are based on MG. Additionally, those devices do not make use of ready-to-use mixes, thus making them potentially less applicable to POC settings. Moreover, we have demonstrated that the SMART-LAMP is applicable, without a specific optimization, for the detection of a number of different pathogens using a single positivity threshold for all the assays, hence providing straightforward applications for new diagnostics. Regarding SARS-CoV-2 RNA detection in clinical samples, the platform presented by Papadakis et al. [56] showed a 97% sensitivity and 100% specificity when analyzing a total of 89 samples. On the other hand, Diaz et al. [55] used the commercial BioRanger diagnostic platform modified for colorimetric LAMP for COVID-19 testing in a limited number of simulated samples spiked with either synthetic RNA or inactivated SARS-CoV-2 virus (*n* = 20; 10 positives and 10 negatives), correctly detecting the 10 positive samples and 9/10 of the negatives. In both studies, the results obtained were comparable with those yielded by our SMART-LAMP device.

We acknowledge the limitations of our study. First, there was a lack of standardization in the manufacturing of the devices, which increased the production complexity and made the manufacturing process labor intensive. In this regard, the limitations presented by other POCT approaches were not remedied [57]. Additionally, only moderate correlation was achieved between RT-qPCR results and SMART-LAMP results, but this was not caused by the desiccation protocol, as correlation values were highly similar between fresh-RT-qLAMP and dry-RT-qLAMP assays (0.75 vs. 0.67, respectively). Thus, the real-time measurements of amplification obtained with the SMART-LAMP were less precise. Considering the results obtained for sensitivity measurements, where correlation values were much higher (R ranging 0.82 to 0.99), we could expect that triplicate measurements would noticeably improve correlation. Finally, one of the main challenges when converting laboratory molecular assays into POC tests was the need to extract nucleic acid as the first step of sample preparation and then to perform the LAMP reaction. However, this could be solved by combining the SMART-LAMP with a number of POC nucleic acid extraction technologies already described that have demonstrated applicability and robustness for the isolation of high-quality nucleic acid from complex raw human samples, including blood, saliva, sputum, nasal swabs, and urine [58].

## 4. Conclusions

The task of bringing affordable molecular diagnostics to the field has been hindering public health improvement for many years [1]. Nevertheless, the increased sensitivity and specificity of molecular methods is needed to address diagnostic challenges in many resource-limited areas. In recent years, the medical field has focused on the development of microfluidic POCT molecular diagnostics. The approaches present numerous advantages, from sample-to-answer design to high sensitivity, specificity or reduced volume [59]. However, some disadvantages must also be highlighted: limited automation selectivity and stability of sensing moduli, as well as poor scalability and clinical validation [28]. Considering the REASSURED criteria [8] as the benchmark to assess POCT, the SMART-LAMP is a real-time connected, affordable solution for sensitive and specific NAAT analysis. It is based on LAMP technology, which has already largely proven its speed and robustness [12,60]. Relying on ready-to-use reaction mixes and a simple smartphone app interface, it is user-friendly and highly deliverable. More extensive studies are required for clinical application and validation; however, this proposal has the potential to become a valuable alternative to the currently available diagnostic options and could be easily adapted to the diagnosis of many other infectious diseases.

## 5. Patents

The SMART-LAMP device is protected under a Utility Model granted by the Spanish Ministry of Energy, Tourism and Digital Agenda, with registry number U202032679, with authors Juan García-Bernalt Diego, Pedro Fernández-Soto, Moncef Belhassen-García, Antonio Muro and Juan M. Corchado Rodríguez listed as inventors.

## Data Availability

The authors declare that data related to this research are available from the authors upon reasonable request.

## Supplementary Materials

The following supporting information can be downloaded at: https://www.mdpi.com/article/10.3390/bios12060424/s1, Figure S1: Single-line electronic circuit sketches of the different modules of the SMART-LAMP; Figure S2: Printed Circuit Board (PCB) of SMART-LAMP; Figure S3: SMART-LAMP app screen workflow; Figure S4: Comparison of fresh-LAMP and dry-LAMP results using malachite green and the effect of storage; Table S1: List of components of SMART-LAMP.
