# Supplementary material for: SMART-LAMP: A Smartphone-Operated Handheld Device for Real-Time Colorimetric Point-of-Care Diagnosis of Infectious Diseases via Loop-Mediated Isothermal Amplification
Source: Biosensors (Basel). 2022 Jun 16;12(6):424. doi: 10.3390/bios12060424 (PMC9221248; doi:10.3390/bios12060424)
Supplement: Supplementary file 1 [file biosensors-12-00424-s001.zip › biosensors-1757728-supplementary.pdf]

SUPPLEMENTARY MATERIAL

SMART-LAMP: A smartphone-operated handheld device for real-time colorimetric point-of-care diagnosis of infectious diseases via loop-mediated isothermal amplification

Juan García-Bernalt Diego[1], Pedro Fernández-Soto[1*], Sergio Márquez Sánchez[2,3], Daniel Santos Santos[2], Begoña Febrer-Sendra, Beatriz Crego-Vicente, Juan Luis Muñoz-Bellido[4], Moncef Belhassen-García[5], Juan M. Corchado Rodríguez[2,3] and Antonio Muro[1].

[1]Infectious and Tropical Diseases Research Group (e-INTRO), Biomedical Research Institute of Salamanca-Research Centre for Tropical Diseases at the University of Salamanca (IBSAL-CIETUS), Faculty of Pharmacy, University of Salamanca, 37007 Salamanca, Spain.

[2] BISITE Research Group, University of Salamanca. Calle Espejo s/n. Edificio Multiusos I+D+i, 37007, Salamanca, Spain.

[3]Air Institute, IoT Digital Innovation Hub (Spain), 37188 Salamanca, Spain.

[4]Microbiology and Parasitology Service, Complejo Asistencial Universitario de Salamanca, University of Salamanca, 37007 Salamanca, Spain.

[5]Internal Medicine Service, Infectious Diseases Section, Complejo Asistencial Universitario de Salamanca, University of Salamanca, 37007 Salamanca, Spain.

*Corresponding authors: pfsoto@usal.es (Telephone: +34 677596173 Ext. 6861)/Antonio Muro: ama@usal.es

CONTENTS

Figure S1. Single-line electronic circuit sketches of the different modules of the SMART-LAMP…………………………………………………………………… **3**

Figure S2. Printed Circuit Board (PCB) of SMART-LAMP……………………... **4**

Figure S3. SMART-LAMP app screen workflow………………………………... **5**

Figure S4. Comparison of fresh-LAMP and dry-LAMP results using malachite green and the effect of storage…………………………………………………….. **6**

Table S1. List of components of SMART-LAMP………………………………... **7**

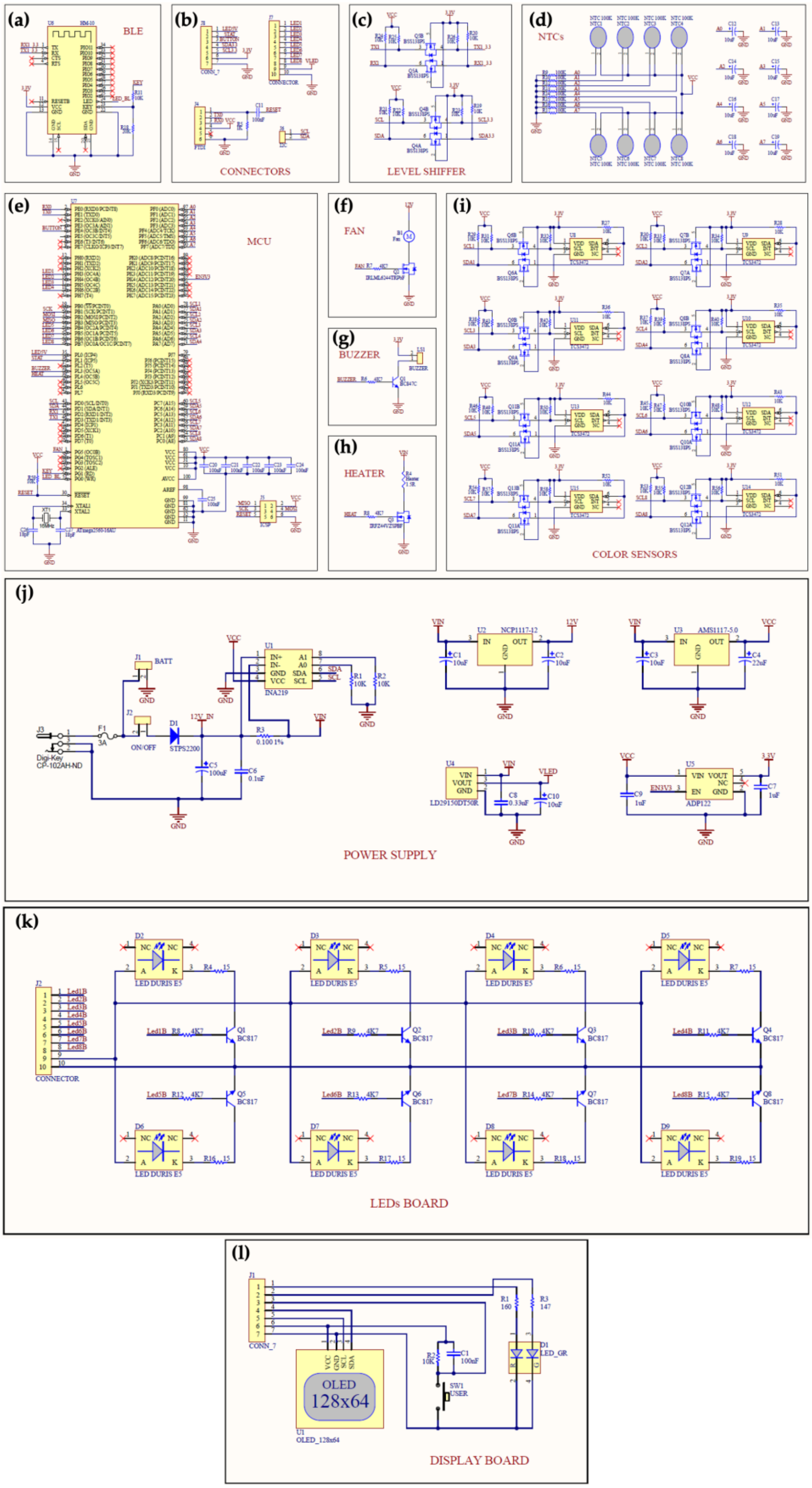

Figure S1. Single-line electronic circuit sketches of the different modules of the SMART-LAMP: **(a)** Bluetooth Low Energy Circuit (BLE); **(b)** Connectors; **(c)** Level Shiffer; **(d)** NTC Thermistors; **(e)** Microcontroller Unit (MCU); **(f)** Fan; **(g)** Buzzer; **(h)** Heater; **(i)** Color sensors; **(j)** Power supply; **(k)** LEDs Board; **(i)** Display Board.

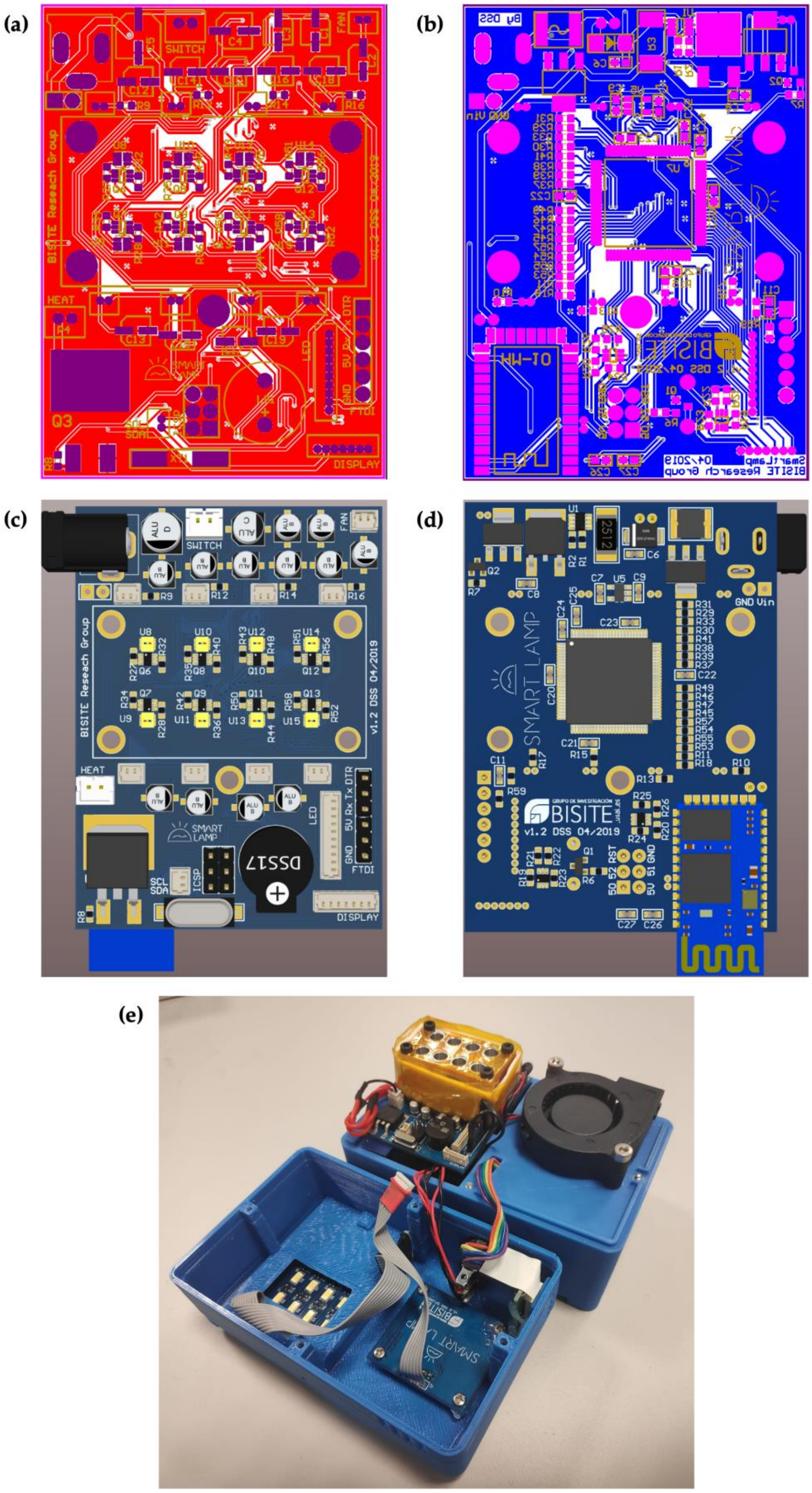

Figure S2. Printed Circuit Board (PCB) of SMART-LAMP: **(a)** Upper face of the PCB; **(b)** Bottom face of the PCB; **(c)** 3D render of the upper face of the PCB; **(d)** 3D render of the bottom face of the PCB; **(e)** Interior of the SMART-LAMP device.

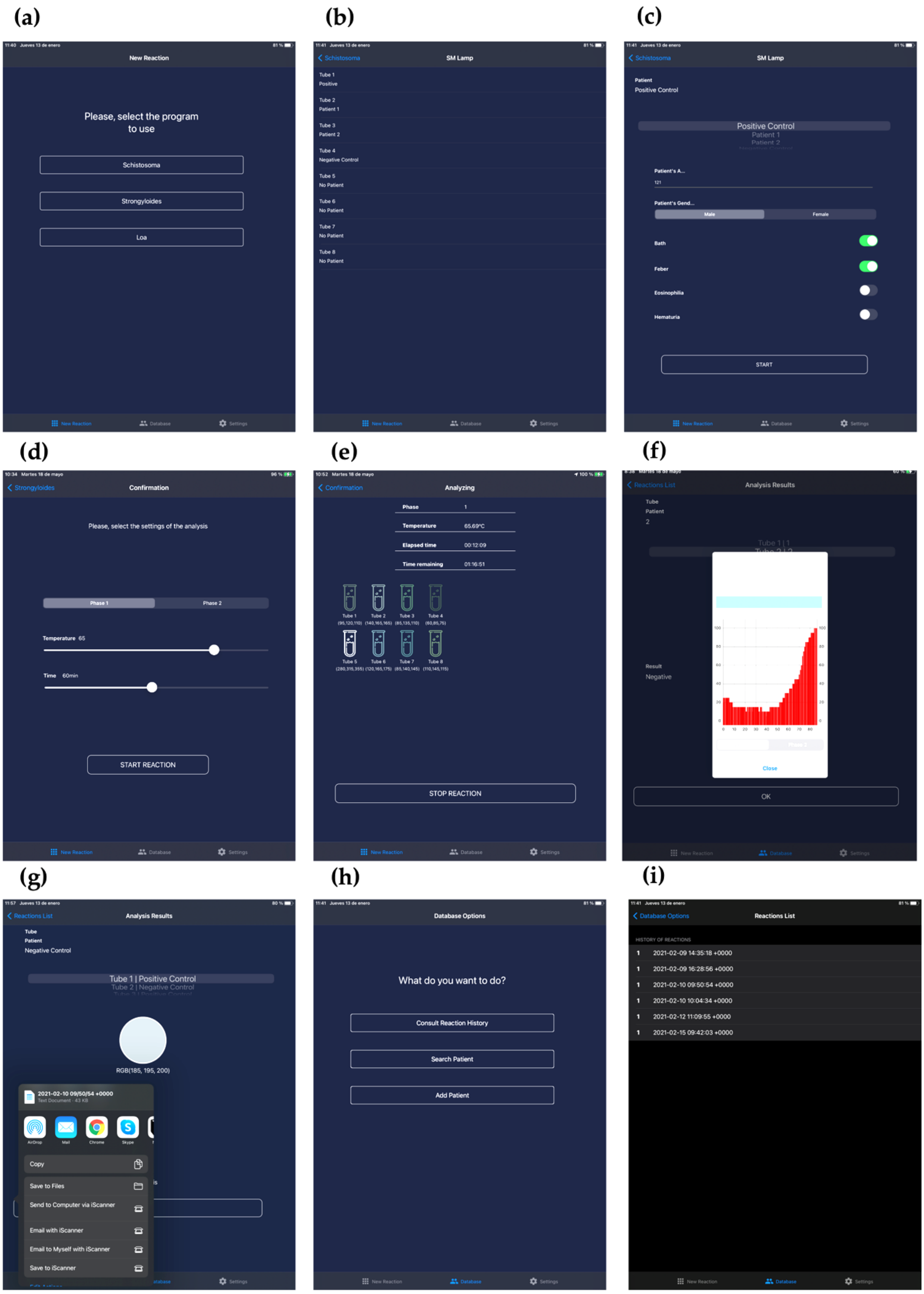

Figure S3. SMART-LAMP app screen workflow: **(a)** Initial screen for the selection of the scheduled reaction conditions; **(b)** Selection of the samples to be analyzed and the position of each sample in the device; **(c)** Screen to add relevant information that has been acquired during sample collection; **(d)** Modification of reaction conditions in terms of temperature and time; **(e)** Real-time visualization of the reaction progress; **(f)** Real-time results for one sample, visualized at the end of the reaction; **(g)** .csv file can be exported at the end of the reaction. (h) Screen to access patient database and previous reactions; **(i)** Past reactions database.

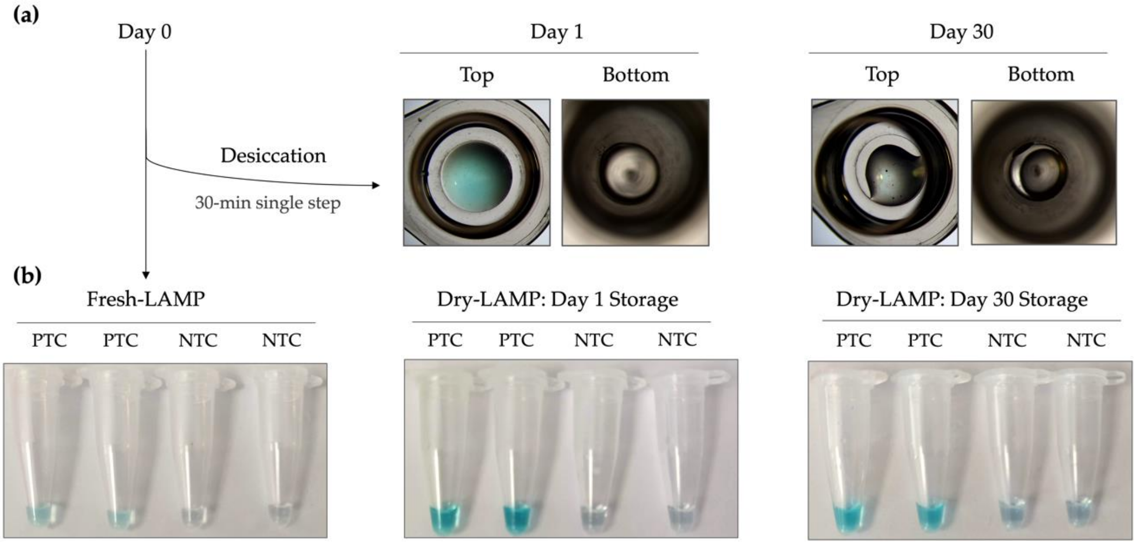

Figure S4. Comparison of fresh-LAMP and dry-LAMP results using malachite green and the effect of storage: **(a)** A view under a magnifying glass of the top (left) and bottom (right) partial mix pellets at day 1 and 30 post-desiccation; **(b)** Colorimetric change of 0.008% w/v MG between positive (PTC) and negative (NTC) results for a LAMP assay targeting *Schistosoma haematobium* as example, using fresh-LAMP and dry-LAMP at day 1 and 30 post-desiccation.

Table S1. List of components of SMART-LAMP.

Modules	Component	Quantity
**Main Board**	ATmega2560-16AU MCU	1
	HM-10 Bluetooth module	1
	TCS3472 RGB Sensor	8
	NTC Resistor 100K	2
	SMD Resistor 100K 0603	2
	Electrolytic Capacitor 10uF 16V	6
	INA219 Sensor	1
	SMD Resistor 0,1 ohms 1% 2512	1
	Electrolytic Capacitor 100uF 16V	1
	Regulator NCP1117-12	1
	Regulator AMS1117-5.0	1
	Regulator LD29150DT50R	1
	Regulator ADP122	1
	Mosfet BSS138PS	10
	Mosfet IRLML6244TRPbF	1
	Mosfet IRFZ44VZ	1
	BC847C Transistor	1
	SMD Resistor 10K 0603	44
	Ceramic Capacitor 100nF 0603	8
	Ceramic Capacitor 1uF 0603	2
	16MHz crystal	1
	Ceramic Capacitor 18 pF 0603	2
	SMD Resistor 4K7 0603	3
	Electrolytic Capacitor 22uF 16V	1
	Ceramic Capacitor 330nF 0603	1
	3A fuse	1
	STPS2200 diode	1
	PicoBlade PCB Header 10 Circuits	1
	PicoBlade PCB Header 7 Circuits	1
	PicoBlade Receptacle Crimp Housing 10 Circuits	1
	PicoBlade Receptacle Crimp Housing 7 Circuits	1
	PicoBlade Female Crimp Terminal	19
	Active buzzer	1
	6x1 male pin header	1
	3x2 male pin header	1
	PicoBlade PCB Header 2 Circuits	1
	JTS-PHX 2-pin connector female	2
	JTS-PHX 2-pin connector male	2
	12V Radial Fan	1
	FTDI module	1
	Conector DC Hembra	1
	Power Switch	1

**Display Board**	OLED Display 128x64 I2C	1
	SMD Button	1
	Ceramic Capacitor 100nF 0603	1
	SMD Resistor 10K 0603	1
	SMD Resistor 147 ohms 0603	1
	SMD Resistor 160 ohms 0603	1
	Bicolor SMD LED	1
	PicoBlade Female Crimp Terminal	7
	PicoBlade PCB Header 7 Circuits	1
	PicoBlade Receptacle Crimp Housing 7 Circuits	1
**LED Board**	LED Duris E5 SMD	8
	BC817 Transistor	8
	SMD Resistor 4K7 0603	8
	SMD Resistor 15 ohms 0603	8
	PicoBlade Female Crimp Terminal	10
	PicoBlade PCB Header 10 Circuits	1
	PicoBlade Receptacle Crimp Housing 10 Circuits	1
**Heater**	Aluminum block	1
	Polyamide heaters	2
	Thermal insulation cm2	36
**Case**	PLA gr	416
	Magnets	6
	Screws	20
